# The Structure of Mental Elasticity Education for Children in Plight Using Deep Learning

**DOI:** 10.3389/fpsyg.2021.766658

**Published:** 2022-02-22

**Authors:** Xuanlu Sun, Xiaoyang Yang

**Affiliations:** ^1^School of Humanities and Social Science, Xi'an Jiaotong University, Xi'an, China; ^2^School of Economics and Finance, Xi'an Jiaotong University, Xi'an, China

**Keywords:** the plight of children, resilience, self-efficacy, attachment relationship, psychological anxiety, deep learning

## Abstract

The purpose is to solve the problem that the current research on the impact of the microstructure of mental elasticity and its constituent factors on the development of the mental elasticity of children is not comprehensive, and the traditional artificial analysis method of mental problems has strong subjectivity and low accuracy. First, the structural equation model is used to study the microstructure of poor children's mental elasticity, and to explore the structural relationship and functional path between the mental elasticity of children and the self-efficacy of their mental health, psychological anxiety, and attachment. Second, a prediction model of mental problems of children in plight based on the backpropagation neural network (BPNN) is constructed. Finally, middle schools in the representative areas of Northwest China are selected as the research unit. The relevant research data are collected by issuing questionnaires, and the data set is constructed to verify the performance of the model. The experimental results show that the average prediction errors of the BPNN model and the support vector regression (SVR) model are 1.87 and 5.4, respectively. The error of BPNN is 65.4% lower than that of SVR, so BPNN has a better performance. The prediction results of the test set show that the actual error and the relative error of the BPNN model are controlled in the range of 0.01, and the prediction accuracy is high. The structural equation model has a high fitting degree. The results of the questionnaire analysis show that attachment, self-efficacy, and psychological anxiety exert a significant direct impact on mental elasticity. This exploration aims to conduct a micro investigation on the relationship among the three core variables (attachment, self-efficacy, and mental health) in the resilience research of children in plight, and analyze their resilience, to provide a theoretical basis for the resilience intervention design of vulnerable groups.

## Introduction

The Chinese government has adhered to incorporating poverty alleviation into the overall plan of the Five-Sphere Integrated Plan and the Four-Pronged Comprehensive Strategy so as to realize the strategic goal of Chinese national rejuvenation. Since the 19th CPC National Congress, targeted poverty alleviation has become one of the three campaigns. Social transformation also includes multiple vulnerable groups, such as orphans and migrant children, many of whom experience various difficulties caused by inequality, vulnerability, and mobility (Bosk et al., 2017). Children in difficult situations include those who are in plight in terms of living, education, and medical treatment due to poverty, those who have difficulty in recovery, nursing care, social involvement caused by disabilities, and those who suffer from threats or harm to personal safety like maltreatment, abandonment, unlawful infringement, and unintentional injury as a result of absent or improper guardianship. When children face difficulties, personal-psychological recovery ability is the core factor that affects personal enthusiasm and subjective initiative. Those with strong recovery ability can often improve their current difficult environment faster. Hence, how they should deal with the difficult situation to develop themselves, and what kind of welfare services can be conducive to their adversity response and adaptation process are the core topics of resilience research.

Mandiü and Pavloviü (2020) pointed out that resilience is a positive adaptation after a stressful situation. Resilience represents the mechanism for coping with and overcoming difficult experiences, that is, a person's ability to successfully adapt to changes, resist the negative effects of stressors, and avoid major dysfunction. It also represents the ability to return to what is previously called “normal” or health after trauma, accident, tragedy, or disease. The higher the resilience is, the lower the vulnerability and the disease risk are. Mental resilience is based on individual differences of different children in adversity adaptation. It mainly refers to the ability of individuals to withstand high-level destructive changes and show as few bad behaviors as possible; it is also the ability of individuals to recover from negative experiences and flexibly adapt to the changing external environment. Zheng et al. (2020) collected a sample of 302 early, middle, and late Italian adolescents from the national junior middle school and senior high school in Sicily, Italy, and verified the relationship between resilience factors and life skill self-efficacy. The results show that there is a strong or medium correlation between resilience factors and perceived self-efficacy in the analyzed fields. However, due to its black-box features, the inner structure of resilience still deserves further exploration. Early studies in China on resilience intervention started from the imitation of overseas intervention projects. For instance, Hong Kong's “The Sky of Growth” project for young teenagers was first implemented in Shanghai (Yang et al., 2020), and then the “Resilient Child Star” project, directed by Professor Peng Huamin, received unanimous recognition from scholars because of its interventional thinking from a strength perspective. However, the quantitative comparisons of the intervention effects of these two projects were both unsatisfactory.

The above research results show that mental elasticity is crucial for children's healthy growth and good adaptation. The research on the influencing factors of mental elasticity will help to improve the quality of children's psychotherapy. However, at present, there are few and immature studies on the impact of the microstructure and constituent factors of mental elasticity on the development of children's mental elasticity. Moreover, the previous prediction of children's mental problems was usually carried out by manual analysis, which had more subjective factors and insufficient accuracy. Based on the above problems, first, the structural equation model is used to study the microstructure of poor children's mental elasticity, and to explore the structural relationship and functional path between children's mental elasticity and the self-efficacy of their mental health, psychological anxiety, and attachment. Then, a prediction model of the mental problems of the children in plight based on the backpropagation neural network (BPNN) is constructed. Finally, middle schools in the representative areas of Northwest China are selected as the research unit. The relevant information of children in plight is collected and summarized by issuing questionnaires; by calculating the weight of the factors affecting the mental problems of the children and ranking them, the first 10 factors are finally selected, and all the data corresponding to these 10 factors are summarized to build a dataset to verify the performance of the model. This exploration is to conduct a micro investigation on the relationship among the three core variables (attachment, self-efficacy, and mental health) in the resilience research of children in plight and analyze their resilience to provide a theoretical basis for the resilience interventional design of vulnerable groups.

## Literature Review

### Children in Plight and Resilience

The city has become impetuous with hubbub due to the rapid progress of society and economy and the construction of urbanization. Adults and children bear various psychological pressures and negative emotions. In such an environment, the pressure that has not been removed in time will inevitably produce the accumulation of negative emotions and greatly affect work, study, and physical and mental health. Generally, adults have their own ways to get some relief from stress. In short, human beings maintain the balance of the heart and the brain by inputting and outputting information. Under normal circumstances, human input will not appear abnormal because the input is conducted through the five senses. However, in such an impetuous living environment, it is relatively difficult to rely on the natural way to output strong negative emotions and unconscious emotions.

Chinese scholars focus on children in plight from the perspectives of psychological development, social stability, protection of rights, and other aspects, mainly reflected in psychology, sociology, and law-related disciplines. There are few studies on the specific problems of children in plight, which may be due to the existence of their plight itself. In order to avoid misanticipating the possible results of children's plight on their future development, some studies have put forward safety policies and measures. Studies on the specific problems of children in plight, such as classified security of children in plight (Wu and Zhou, 2018), high-risk adolescents (Edalati and Conrod, 2019), social support for children in plight (Alon, 2019), and service strategies for children in plight (Li, 2020), have a premise that children in plight will inevitably lead to poor development or damage to social equity. Zheng et al. (2020) showed that children need more timely help and guidance than adults because they are in the development stage of language and autonomous behavior.

The definition, connotation, and extension of children in difficulty are a controversial issue in academic circles. In academic research, scholars use a broader concept, which refers to all children whose survival and development are in trouble because of poverty, disease, accident, neglect, and family abuse (Sobol-Kwapińska et al., 2020). The early concept of children in plight comes from the description in child welfare policy of international organizations, such as “children living in extreme distress (Hoyt et al., 1997).” Moreover, terms such as “special needs”, “special hardship”, “social disadvantage”, “most vulnerable”, and “in need of help” vary from time to time, which express the life situation of children in difficulties (Benn et al., 2020). To break the inter-generational transmission of poverty and enhance the use of National Public Funds Return in developing welfare services for children in plight, Western scholars often intervene at the family level, focusing on the All-inclusive Welfare policy which supports improvements to family care. Chinese researchers primarily focus on the social transformation and the transformation of family structure and function. They discussed that social transformation has made the number of orphans, disabled children, left behind children, poor children, divorced families, street children, prisoners, injured children, sick children and AIDS orphans increasing. There was even a time only children were included as a consequence of social transformation.

Based on the above definition of children in plight, children in plight are classified based on three levels: family, individual, and social reasons. There are three types of children in an individual dilemma: seriously ill children, disabled children, and children with psychological disorders; the children in a difficult situation based on family predicament also include three categories: (1) single-parent family children, left-behind children, and abandoned children due to the lack of parental monitoring function in the family; (2) children whose parents are unable to support them due to severe illness and disability; (3) children whose parents are divorced or dead, and children who are not in maintenance or guardianship. Children with social difficulties include street children, children infected with AIDS, and children whose parents have served a long-term sentence or detoxification.

### Three Core Variables: Attachment, Self-Efficacy, and Mental Health

Resilience research focused on variables that show that the adaptive development of children in plight can influence children's physical health, temperament and their psychological recognition, emotion, and abilities, such as trust in others (Arthur and Reynolds, 2010), good intelligence, self-respect (Silverthorn et al., 2017), a sense of responsibility, humor, independence, future orientation, positive tendency, attribution mode of internal control, and religious belief. The parenting quality, prosocial behaviors, parent–child relationships, and emotional atmosphere in children's families all play a supportive and regulatory role in the adaptive development of children.

Recently, the concept of self-efficacy has become a research hotspot in multiple disciplines, such as sociology, psychology, nursing, and education, and it is common in masters and doctoral theses in different disciplines (Wiedenhofer and Koch, 2017). It is still a crucial index to evaluate individual positive self-efficacy, which can be divided into self-efficacy and special self-efficacy (such as job-seeking self-efficacy, self-management self-efficacy, and professional self-efficacy) (Madokoro et al., 2021), or classified according to the evaluation of positive cognition of self-ability (such as mathematical self-efficacy) of different disciplines (Khedhaouria et al., 2017). Moreover, it is closely related to personal social adaptation, job performance, leadership, creativity, and subjective well-being. Crucial indices of individual adaptation results and optimistic prediction of performance level can be developed through appropriate training (Murphy et al., 2017). Self-esteem, sense of responsibility, positive tendency, internal control, future orientation, and other factors related to self-efficacy are the key factors to study resilience under trait theory. Hence, it is natural to regard self-efficacy as an indispensable index in the training of resilience (Sagone et al., 2020).

Affecting children's interpersonal relationships and social adaptation results can positively predict the mental health of orphans (Yujia et al., 2017). The research on the resilience of left-behind children shows that the attachment relationship between left-behind children and adults can compensate for their resilience (Tripa et al., 2021). The most common subject of the research on attachment is the mediator variable, which serves as a family functioning and a result of children's adaptation, and explores the intervening functions of attachment on various adaptation results. For instance, the research on high school students reveals that parent–child attachment is highly relevant to the significance of children's lives and may impact college students in developing a sense of self-worth and safety (Yoder et al., 2018). Father–child attachment has a prominent intervening effect on family function and the emotional health of the child (Khambati et al., 2018) and is closely related to family function, school adjustment, interpersonal relationship, and withdrawal behaviors (Zou and Wu, 2020). When the attachment relationship exerts a long-term effect on individual growth, it may influence children's recognition and emotion after becoming adults (Ma and Zhang, 2020).

The father-child attachment will influence the mother's gate-keeping behaviors, improving or hindering the binary interaction mode between mother, father, and child (Wall, 2018). Parent–child attachment affects children's social adaptation results under different situations and has an evident intervening effect among various relationships, predicting the mental health of specific children (Zhang et al., 2019). Parent-child attachment is also connected with how family members interact and influence the interaction mechanism among family members (Tozer et al., 2019). Hence, it is very suitable to use parent-child attachment as an influential structural factor of resilience to develop and cultivate resilience.

Mental health is the result of good adaptation at the individual level. It is the common pursuit of the spiritual world after people obtain a rich material life, which is the most extensive research field. Present mental health research is characterized by the following points. (1) Covering almost all kinds of groups. As people have universal mental health demands, this exploration covers nearly all groups of people; (2) Diverse and complex situations. This exploration involves human behaviors under various contexts, such as mental healthcare in workplaces and mental health responses during the epidemic; (3) Correlating with adverse events, like the risks and stress from difficult situations. Mental health research mostly follows the underlying hypothesis that terrible environmental events will harm mental health; (4) Broad research levels. This exploration involves the evaluations, intervention, cultivation, and internal and external mental health action mechanisms. Meanwhile, mental health is a dependent variable to research individual adaptation results and an independent variable to analyze other related personality traits and adaptation conditions. Given the above analysis, mental health and individual adaptation are mutually interactive, and both of them serve as a vital evaluation indicator of good resistance in the consequence-based theory of resilience.

There are different views on the research of mental health and mental resilience. For example, mental resilience training can promote mental health (Gregoire et al., 2018); and mental resilience can mediate between social support and emotional response (Khawaja et al., 2017). Resilience studies have also defined resilience by treating mental health as the result of resilience (Fellmeth et al., 2018).

In conclusion, the attachment relationship between children and adults, children's self-efficacy, and mental health level are all crucial for resilience research. Therefore, exploring and analyzing the internal relations among the three factors mentioned above can help identify their intervention effect in resilience research and offer a theoretical foundation for resilience cultivation research.

## Research Method

### Prediction of Mental Health Problems of Children in Plight Based on Deep Learning

**Figure 1:**
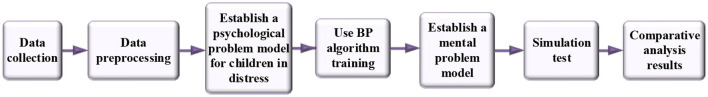
Research ideas of the mental health of children in plight based on deep learning.

The main factors affecting the mental problems of children in plight are taken as the research subject. The basic personal information, social support, and other factors are added into the data, and the BPNN is adopted to build the mental problem prediction model. The influential data of mental problems of children in plight are obtained by learning and training the various influencing factors. Then, the complete results of training are analyzed to make the prediction model closer to the actual characteristics of the questionnaire samples. Figure 1 displays the research idea.

### The Research Paradigm Based on Variables

The concept of resilience originates from a phenomenological finding: among those in difficult situations, some can adapt well. However, scholars have proposed multiple hypotheses for why this is the case. The concept of resilience has been explained by trait theory, consequence-based theory, process theory, and capacity theory. Trait theory believes that resilience is unique to others, making people survive in risk and adversity. Consequence-based theory regards resilience as a result of the excellent adaptation of individuals after experiencing difficulties. Process theory holds that individual resilience is the result of the interaction between internal and external protective factors and dynamic risk factors.

Capacity theory considers resilience as a capacity, making individuals overcome difficulty and adapt well to adversity. Regardless of the conceptual hypothesis of resilience, the resilience process consists of two core factors: protect factor and risk factor. Then, scholars have made unremitting efforts on the impact of risk and protective factors on the development and cultivation of resilience. Variable-based resilience research is a paradigm to explore the interaction effect between risk and protective factors.

The key to the paradigm of variable-based research is to investigate the source of variables and factors affecting resilience development and predict the process and mode of resilience development to provide theoretical evidence for resilience intervention. The research paradigms include direct relationship graphs, indirect relationship graphs, and interaction models. The direct relationship graph holds that risk factors and protective factors contribute to the adaptation results independently. Furthermore, protective factors make positive contributions to adaptation, while risk factors negatively impact them.

The model assumes that protective factors can overlap with risk factors, and accumulative results can predict individuals' capacity development and adaptation results. The intervention mode for children in plight based on a direct relationship graph can be compensated by increasing the protective factors. Theoretically, as long as children have adequate protective resources, the adverse effects brought by risks can be counterbalanced, and the adaptation results can be maintained at a relatively high level (Whitney and Peterson, 2019). In a direct relationship graph, the adverse effects of risks can be compensated by protective factors. Therefore, Norman Garmezy calls it a compensatory model.

The indirect relationship model suggests that risk factors are not directly related to the outcome of children's psychosocial development, and there may be mediating or indirect effects between them. In the indirect relationship model, the protective factors have a mediating effect on risk, which may be a complete or incomplete intermediary. Under the influence of a complete intermediary, the risk does not directly affect the development of social functions but indirectly they are affected by the transmission of protective factors. For example, parental rearing function on children's psychological development has an indirect mediating effect (Farrelly, 2013). The interaction model is characterized by multiple regulatory factors while risk factors act on individuals. Regulatory factors can change the impact of risk on adaptation results, known as protective factors or susceptibility factors. The two factors have opposite effects on the results of psychological adaptation (Yun and Juvonen, 2020).

The regulatory effects of interaction have two types. The first type focuses on individual characteristics during risk response or the influence of individuals' environment in disadvantaged situations. These factors are always unrelated to risks and exist independently, like individual personalities that can regulate the adverse effects of risks. The second type is the protective factor activated by risk factors, that is, when risks appear, this kind of factor will be activated and perform its protective functions (Peris et al., 2020). For example, once children encounter risks or adversity, their parents' guardianship effects will be activated to protect them from harm.

To study the effects of self-efficacy, attachment, and mental health on resilience, an indirect relationship graph is taken as the basis of the research hypothesis to verify the relationship model between the three variables and resilience.

## The Proposition and Testing of the Structural Equation Model

### The Description of Variables

This exploration has four latent variables, namely psychological anxiety, resilience, attachment, and self-efficacy. The structural equation model is adopted to study the direct effects of the impact of the resilience of children in the plight in the northwest region and the indirect and complex relations among four variables. Psychological anxiety adopts “Yes” and “No” to assign each item, while attachment and self-efficacy are scored from “1” to “5”, which refers to a range from “Totally Agree” to “Totally Disagree,” respectively. Table 1 presents the results of various variables.

**Table 1:** Variable description.

	**N**	**Min**	**Max**	**Mean**	**Standard**
					**deviation**
Stress response efficacy	1,638	5	20	14.54	2.82
Strive for self efficacy	1,638	5	20	11.60	2.90
Study anxiety	1,638	0	10	6.44	2.13
Social anxiety	1,638	0	10	4.43	2.20
Isolation tendency	1,638	0	10	3.08	2.36
Self-accusation tendency	1,638	0	10	6.14	2.39
Allergic tendency	1,638	0	10	6.41	2.03
Physical symptoms	1,638	0	10	4.55	2.35
Terror tendency	1,638	0	10	3.90	2.82
Impulsive tendency	1,638	0	10	3.24	2.42
Escaping	1,637	23.00	112.00	64.40	14.85
Anxiety	1,638	18.00	120.00	64.37	18.38
Inner strength	1,622	20.00	75.00	52.30	8.77
External support strength	1,637	14.00	60.00	39.95	7.52

### The Description of Variables

#### Resilience

Resilience refers to individuals' ability in adversity to effectively cope with and adapt to difficult situations and get recovered from them. The resilience factor structure here is based on the hypothesis of the resilience concept from trait theory and at the same time, focuses on the supportive impacts of an individual's ecosystem on the resilience process. Thus, this survey on resilience uses the teenager resilience scale with 27 entries, which has five content scales, namely the concentration on goals, emotional control, positive cognition, family support, and the assistance on interpersonal relationship, or two dimensions, namely personal power and support power. Higher scores will be a demonstration of better resilience.

Personal power (concentration on goals, emotional control, and positive cognition) and support power (family support and assistance on interpersonal relationships) are taken as the micro measurement indicators of resilience. The Kaiser-Meyer-Olkin (KMO) value of scale is 0.873, the Chi-Square value of Bartlett's Test is 10,589.33, and the significance is at 0.00. These data demonstrate that the scale has good reliability and validity and can be used for factor analysis and model testing.

#### Attachment

The scale of experiences in close relationships inventory (ECR) includes 36 items and is divided into two subscales, with 18 entries in each. Items of odd numbers belong to the subscale of attachment avoidance, while items of even numbers belong to the subscale of attachment anxiety, among which the 15th, 22nd, 25th, 27th, 29th, 33rd, and 35th entries are reversely scored. The scale adopts a 7-level scoring method. Those entries requiring a reverse scoring method are reassigned as new variables. The newly calculated results of the variable are put in the scoring of attachment anxiety and the scale of attachment avoidance to obtain the related two scores.

Higher scores of attachment avoidance mean more anxious experiences of individuals, that is, they will be more worried about others' closeness, thus showing avoidance behaviors toward others' closeness. Higher scores of attachment anxiety indicate individuals' greater willingness to be with others; Therefore, less sense of belonging will make them feel lonely and afraid of being abandoned. The KMO value of scale is 0.907, the Chi-Square value of Bartlett's Test is 15,748.55, and the significance is at 0.00. These data suggest the good reliability and validity of the scale, which are applied to factor analysis and model testing. The attachment relationship between children and adults tends to decide children's choice of coping strategy under pressure. Hypothesis 1 is proposed based on that.

H1: Attachment has direct effects on resilience.

#### Mental Health

The basic definition of mental health is that people's psychological and mental processes are reasonable or normal. The mental health table (MHT) of primary and middle school students for mental health tests is used. Moreover, MHT totals eight subtables, including learning anxiety, anxiety toward others, autistic tendency, self-accusation tendency, allergic tendency, physical symptoms, fear tendency, and impulse tendency. Each subtable has ten entries to test children's mental health conditions better and comprehensively. Higher scores mean more obvious-related symptoms. The KMO value of scale is 0.910, the Chi-Square value of Bartlett's Test is 22,648.4, and the significance is at 0.00. These data suggest the good reliability and validity of the scale, which has been universally applied in academic research nowadays. Mental health is a vital testing indicator of coping with personal stress. Hypothesis 2 is proposed based on that.

H2: Mental health has direct effects on resilience.

#### Self-Efficacy

Self-efficacy is a concept put forward by Bandura in the 1980s. It is believed that self-efficacy, people's belief in the ability to complete a specific behavior or produce a particular result, is a particular self-ability expectation of an individual. Moreover, it is the core of an individual's perception of goal selection and effort and the individual's perception of their self-control ability. It is a kind of self-generating ability that integrates cognitive, social, and behavioral skills. It is also the judgment of people's behavior process and ability to achieve specific goals to be implemented in an organization. It is also the belief that affects the ability to control self-life events. Self-efficacy is like self-concept or self-esteem, as all of them describe the subject's phenomenon, taking the subjects' self as a reference to reflect and evaluate themselves. The scale comprises ten entries, which evaluate children's ability to solve problems by themselves, to deal with emergencies and accidents, to stick to and strive for goals, to calmly analyze and handle problems, and to work hard in difficulty.

The overall reliability of the self-efficacy questionnaire has been tested, which is good. The approximative Chi-Square value of Bartlett's Test is 3,318.17, the significance is at 0.00, and the KMO value of scale is 0.862. The test shows that factor analysis is available. The principal component analysis (PCA) approach is adopted to assign characteristic root as a value greater than 1, and the loadings of 10 items are extracted. Each item's loading is greater than the practical value of 0.4, and two factors are extracted precisely. As the items of the first factor are all surrounded by the evaluations on children in plight solving complex problems by their efforts, they are defined as “effort self-efficacy.” The items of the second factor are all surrounded by the evaluations on children's ability to cope with difficulty or accidents under pressure so that they are defined as “stress-coping efficacy.” Table 2 reveals the results of the factor analysis.

**Table 2:** Factor analysis of self-efficacy of children in plight.

**Question**	**Factors**	
	**1**	**2**
1. If I try my best, I can always solve the problem.	0.430	
2. Even if others oppose me, I still have a way to get what I want.		0.622
3. It is easy for me to stick to my ideals and achieve my goals.		0.784
4. I am confident that I can deal with any sudden event effectively.		0.498
5. With my intelligence, I can cope with unexpected situations.		0.683
6. If I make the necessary efforts, I will be able to solve most of the problems.	0.724	
7. I can face difficulties calmly because I trust my ability to deal with problems.	0.700	
8. While faced with a difficult problem, I can usually find several solutions.	0.782	
9. When I am in trouble, I can usually think of some ways to deal with it.	0.727	
10. Whatever happens to me, I can handle it.		0.453

According to the results of factor analysis, the scores of the five entries, the 2nd, 3rd, 4th, 5th, and 10th, are summed to form the “stress-coping efficacy” factor. Likewise, the scores of these five entries, the 1st, 6th, 7th, 8th, and 9th, are summed to form the “efforts self-efficacy” factor. The above mentioned two factors are considered as two observed variables of children's self-efficacy, and the following hypotheses are proposed based on them:

H3: Self-efficacy has direct effects on resilience.

H4: Self-efficacy has mediating effects on the relationship between attachment and resilience.

H5: Self-efficacy has mediating effects on the relationship between mental health and resilience.

#### The Correction of Hypotheses

Since there is controversy about the effects of mental health on resilience, considering the effectiveness of all hypotheses, attachment, mental health, self-efficacy, and other factors are incorporated into the equation of regression for linear regression. The results reveal that only learning anxiety, anxiety toward others, autistic tendency, and impulsive tendency can enter the regression equation among the eight factors of mental health while the others cannot. Besides, the test of the regression coefficient is significant. R^2^, the coefficient of determination of the equation, is 0.413, the adjusted R^2^ is 0.41, and the result of the *F*-test is 141.59. Hence, the equation has passed the test, and it has higher explanatory power. Therefore, these four mental health factors are redefined, namely learning anxiety, anxiety toward others, autistic tendency, and impulsive tendency, as “mental anxiety,” referring to children's mental, anxious, and impulsive behavioral tendencies in school life. Table 3 shows the linear regression model of psychological elasticity of children in distress.

**Table 3:** A linear regression model for resilience of children in plight.

**Model**		**Unstandardized coefficients**		**Standardization coefficient**	**t**	**Significance**
		**B**	**Standard error**	**Beta**		
1	(Constant)	140.483	1.991		70.551	0.000
	Escaping	−0.269	0.020	−0.282	−13.356	0.000
	Anxiety	−0.070	0.017	−0.090	−4.087	0.000
	Impulsive tendency scored	−0.667	0.134	−0.114	−4.985	0.000
	Isolation tendency scored	−1.373	0.135	−0.229	−10.177	0.000
	Social anxiety scored	−0.317	0.159	−0.049	−1.997	0.046
	Study anxiety scored	−0.362	0.148	−0.054	−2.455	0.014
	Stress response efficacy	−0.843	0.113	−0.172	−7.445	0.000
	Strive for self efficacy	−0.444	0.116	−0.088	−3.844	0.000

Based on this, the author corrected Hypotheses 2 and 5 as follows:

H2′: Mental anxiety has direct effects on resilience.

H5′: Self-efficacy has mediating effects on the relationship between mental anxiety and resilience.

### The Structural Equation Models

**Figure 2:**
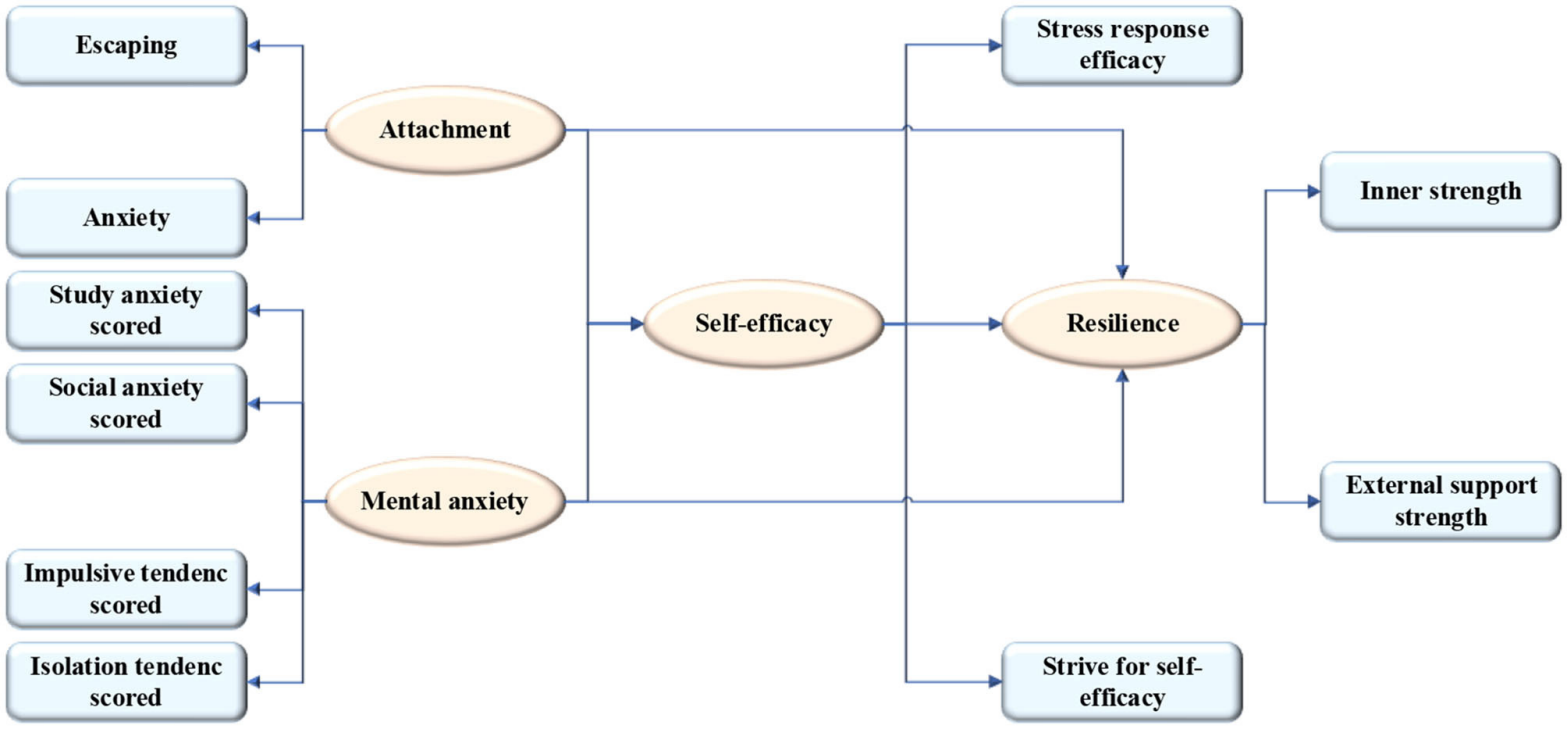
The mediation model of the microstructure of resilience.

Based on all the mentioned hypotheses, an initial model is structured according to the relationship among four latent variables, mental health, self-efficacy, parent–child attachment, and mental elasticity. Figure 2 presents the mediation model of the microstructure of resilience.

### Feature Extraction of Deep Neural Network Data

#### Building BPNN Prediction Model

**Figure 3:**
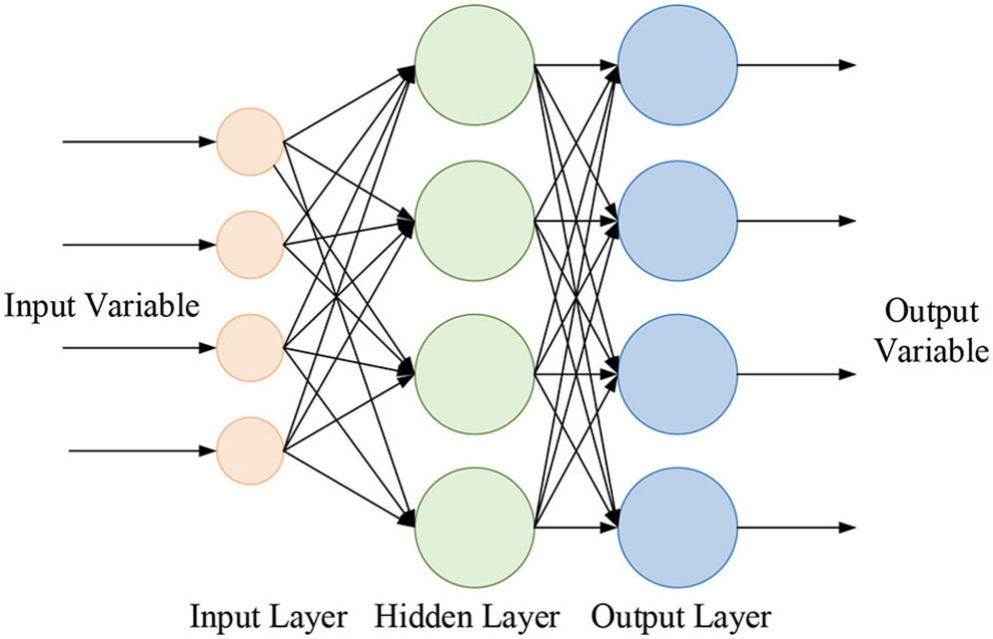
BPNN structure.

The BPNN algorithm is optimized by building the BPNN model. The model is simulated and trained to reach the optimized state according to the internal and external factors that affect the mental problems of children in plight. The BPNN learning model refers to the neural network structure design of the prediction model. The structured data of students' mental health problems (data from training samples) is taken as the input node, and the filtering conditions are established according to the psychological prediction. The number of the middle (hidden) layer, the structure of the node counting model, and the output node are the preliminary expectations for predicting mental problems. Figure 3 presents a schematic of the BPNN.

The process of determining model parameters includes the optimization of network structure, activation function, training times, and learning rate parameters. This exploration mainly predicts the mental problems of children in plight, so the number of nodes of the input layer in BPNN is 10; the number of output neurons is 3, indicating normal psychology, mild mental problems, and severe mental problems, respectively. Therefore, it is only necessary to find the optimal number of hidden layer nodes. The general calculation equation for the number of hidden layer nodes is as follows:


(1) $$\begin{array}{r} M=\sqrt{NL}+\alpha \end{array}$$

M, N, and L are the numbers of nodes in the hidden layer, input layer, and output layer, respectively; α is an integer of [1, 10].

## Experimental Design

### Experimental Environment Setting

MATLAB simulation software is used as the main modeling tool, and EXCEL is taken as the data carrier and standardized processing tool. The combination of the two gives rise to their respective advantages so that the process from the original data to the final result can be successful, which greatly improves efficiency. Excel Link is a software plug-in used to integrate EXCEL and MATLAB. Through the link between MATLAB and EXCEL, users can use the data processing and graphics processing functions of MATLAB to carry out relevant operations in the EXCEL workspace; meanwhile, EXCEL ensures the data exchange and synchronous update of MATLAB and EXCEL workspace. Excel Link can be used without leaving the EXCEL environment. It is only necessary to call the MATLAB function directly in the workspace or macro operation of EXCEL.

### Data Sources and Sample Descriptions

The representativeness of samples in quantitative research determines the explanatory power of data on social phenomena. The data used here are derived from the survey data from a 2019 National Social Science project named *The Research on the Resilience Development and Welfare Services of Children in Difficulty in Northwest China*. Stratified cluster random sampling is used to select the samples for the data survey.

According to the research needs, three representative regions from Northwest China are selected as the survey sample sources: Shaanxi, Gansu, and Ningxia. Three cities are randomly selected among small and medium-sized cities of the previously mentioned province as the school sample sources. According to the convenient connection principle, a middle school (junior and high middle school) is selected and a presurvey is conducted for children in plight. In actual surveys, based on the filing screening result of the sources of students from every school, those children whose families meet the standards of poor households are defined as children in plight, and the others are defined as children not in plight to form a respondent group.

Sample source. Samples from Shaanxi, Gansu, and Ningxia are selected, 1,700 questionnaires are distributed, and 1,638 samples of valid data are obtained, with an effective rate of 96.4%. Respondents are asked to fill in the questionnaire on the spot and investigated one-to-one. The questionnaires are collected on the spot and experimenters host the filling process. Respondents can leave after completing the questionnaire with no blank missed. Experimenters will be in charge of the explanation of the questionnaire. Table 4 displays the primary conditions of the respondents.

**Table 4:** Situation of respondents (*n* = 1,638).

**Variables**	**Value**	**Number of relevant personnel**	**Percentage (%)**
Gender	Male	728	44.4
	Female	910	55.6
Age	10–18	Mean 14.77	Std.1.69
Periods of study	High school	453	27.7
	Middle school	1185	72.3
Region	Shaanxi	342	21.4
	Gansu	463	28.3
	Ningxia	833	50.3
Type of plight	Vagrancy and begging	7	0.6
	Absence of guardianship	105	8.6
	Left behind mobility	90	7.4
	Family violence	17	1.4
	Low-income households	430	35.4
	General poverty	797	65.7
	Special difficulties	162	39.5
Level of plight	Level I	104	8.8
	Level II	726	61.7
	Level III	346	29.4
Family economic status	Extremely poverty	97	5.9
	Poverty	801	49.0
	General condition	727	44.5
	Affluent families	9	0.6
Marital status of parents	In marriage	1353	84.7
	divorce	82	5.1
	remarriage	67	4.2
Siga	Single parent	94	5.9

The questionnaire design includes basic information, types of difficult situations, mental health scale, behavioral problems, personality characteristics, emotional relations, self-efficacy, attachment, resilience scale, and the scale on the information of the left-behind children and their parent-children interaction. Basic information covers the description of subjects' age, gender, school grade, family condition, the people they live with, family economic status, and parents' marital status. Classifying the problematic situations is to differentiate the characteristics of children's difficulties, including roaming in the streets and begging, the shortage of custody, being left-behind and migrant, domestic violence, households receiving subsistence allowances, general poverty, and particular difficulty.

### Selection of Characteristic Factors and Establishment of Dataset

The adjustment results of various attributes and feature factors affecting children's mental problems reflect the mental health status of the children in plight. The weights of these factors are adjusted to make the adjusted results more accurate. Specifically, among the three types of mental problems affecting the internal and external factors, such as personal basic information and social support, massive data are collected to rank the performance attributes, the influence magnitude, the range of weighted influence score equation, and the descending rule of each attribute score. The SCL-90 is employed as a reference. A total of 10 elements are set, each with a different project. The data are processed equally for each factor and Table 5 presents the results.

**Table 5:** Partial results of SQL data processing.

**N1**	**N2**	**N3**	**N4**	**N5**	**N6**	**N7**	**N8**	**N9**	**Others**
1.43	2.05	1.89	1.85	2.07	1.21	2.21	1.07	2.07	2.13
1.77	1.21	2.28	1.11	1.83	1.82	1.94	2.06	2.10	1.33
1.98	1.87	1.63	1.57	1.32	1.63	1.66	1.13	1.19	1.57
1.29	2.52	2.05	1.99	1.01	1.21	1.21	1.75	1.75	1.69
1.73	1.07	1.01	1.02	2.69	1.59	1.53	1.21	1.21	2.22

All the data corresponding to each factor are summarized and the dataset is constructed. The dataset contains 10 sub-datasets, among which 4 are randomly selected as the training set, and the other 6 are used as the test set. When data samples are used for network training, massive small data need to be normalized because the data samples are fragmented, diversified, and are one-dimensional. The normalization equation is as follows.


(2) $$\begin{array}{r} \bar{ai}=\frac{ai-a\;\min}{a\;\max-a\;\min} \end{array}$$

where is the input and output data, *a*max is the maximum value in the data, and *a*min is the minimum value in the data.

### Parameter Optimization and Performance Test Method of BPNN Model

The numbers of nodes in the hidden layers are set to 5 and 11, respectively. With the root mean square error (RMSE), mean absolute error (MAE), and mean square error (MSE) of the model in the training process as indicators, validation experiments are conducted to determine the optimal number of hidden layer nodes. The equation reads:


(3) $$\begin{array}{r} RMSE=\sqrt{\frac{1}{n}\sum_{i=1}^{n}{\left({ŷ}_{i}-y_{i}\right)}^{2}} \end{array}$$

(4) $$\begin{array}{r} MAE=\frac{1}{n}\sum_{i=1}^{n}|{ŷ}_{i}-y_{i}| \end{array}$$

(5) $$\begin{array}{r} MSE=\frac{1}{n}\sum_{i=1}^{n}{\left({ŷ}_{i}-y_{i}\right)}^{2} \end{array}$$

ŷ_*i*_: predicted value; *y*_*i*_: actual value; n: total number of samples.

Sigmoid and tanh are selected as activation functions for comparative experiments to determine which one is better. A training cycle is followed to completely traverse the dataset once, and it is not advisable to train too many times, or too few. Therefore, the best amount of training can be found by training for different amounts. In the training process, less small learning rate will cause the training to stop before finding the best parameters. Many large learning rates may cause oscillation or divergence, resulting in the failure to find the best advantages. Therefore, the optimal learning rate parameters must be found. Support vector regression (SVR) [38] prediction algorithm is selected as the control. Based on the test set, BPNN and SVR models are adopted to predict the mental problems of children in plight. RMSE, MAE, and MSE are calculated, and the prediction results are compared and analyzed to explore the prediction performance of this model. The penalty coefficient of the SVR model is set to 0.5, and the radial basis function is selected as the kernel function.

### Experimental Result

#### BPNN Network Parameter Optimization and Performance Test Results

**Figure 4:**
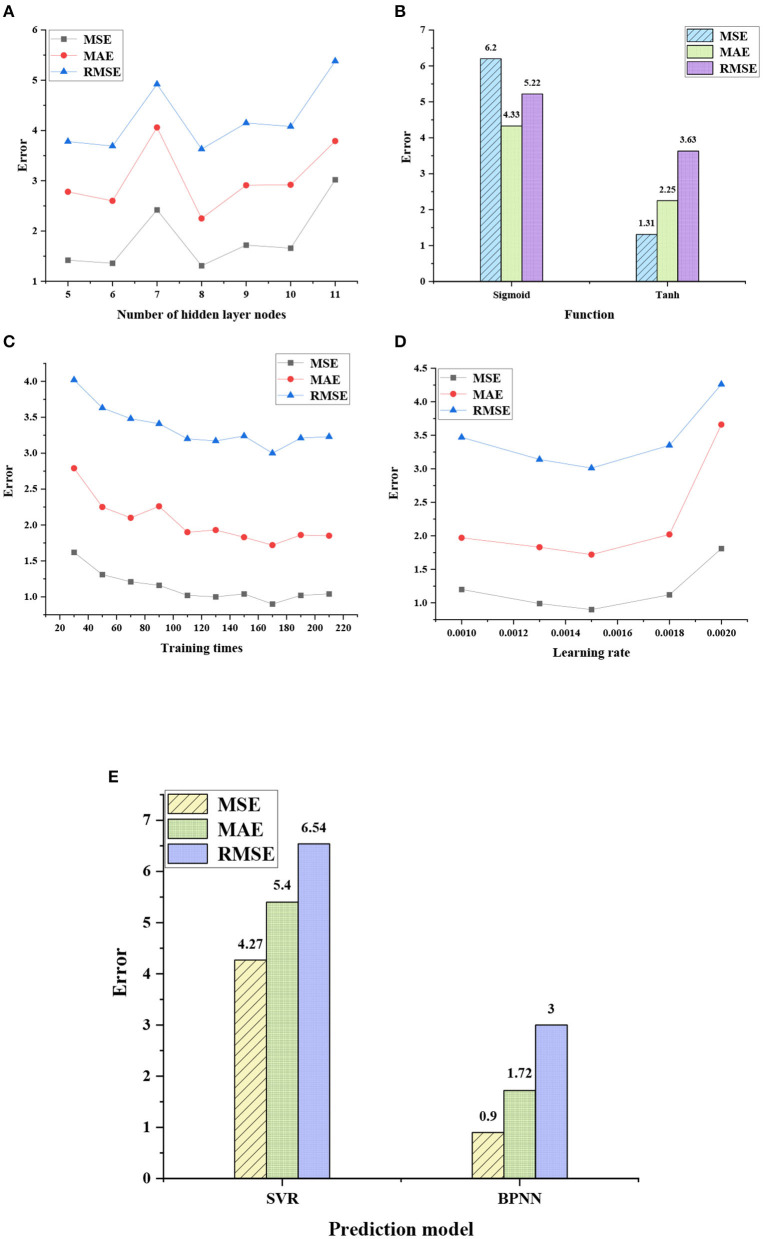
Performance test results of the model under different parameters. **(A)** Optimization result of nodes in the hidden layer; **(B)** Optimization result of activation function; **(C)** Optimization result of training times; **(D)** Optimization result of learning rate; and **(E)** Comparison of prediction performance of the two models.

Figure 4 shows the optimization results of parameters in different hidden layer nodes, activation functions, training times, and learning rates, as well as the performance test results of BPNN and SVR models.

The comparison of the prediction errors of the two models shows that the average prediction error of the SVR model is 5.4 and that of the BPNN model is 1.87, which is 65.4% lower than that of the SVR model. The performance of the prediction model under different parameter settings shows that the model performance is the best when the number of hidden layer nodes is 8, the activation function is the tanh function, the training count is 170, and the learning rate is 0.0015. Therefore, Table 6 is the best parameter setting of the network.

**Table 6:** Summary of BPNN optimal parameters.

**Parameters**	**Value**
Number of nodes in the input layer	10
Number of nodes in the output layer	3
Number of nodes in the hidden layer	8
Activation function	Tanh
Training times	170
Learning rate	0.0015

#### The Prediction Result Display of the BPNN Model

**Figure 5:**
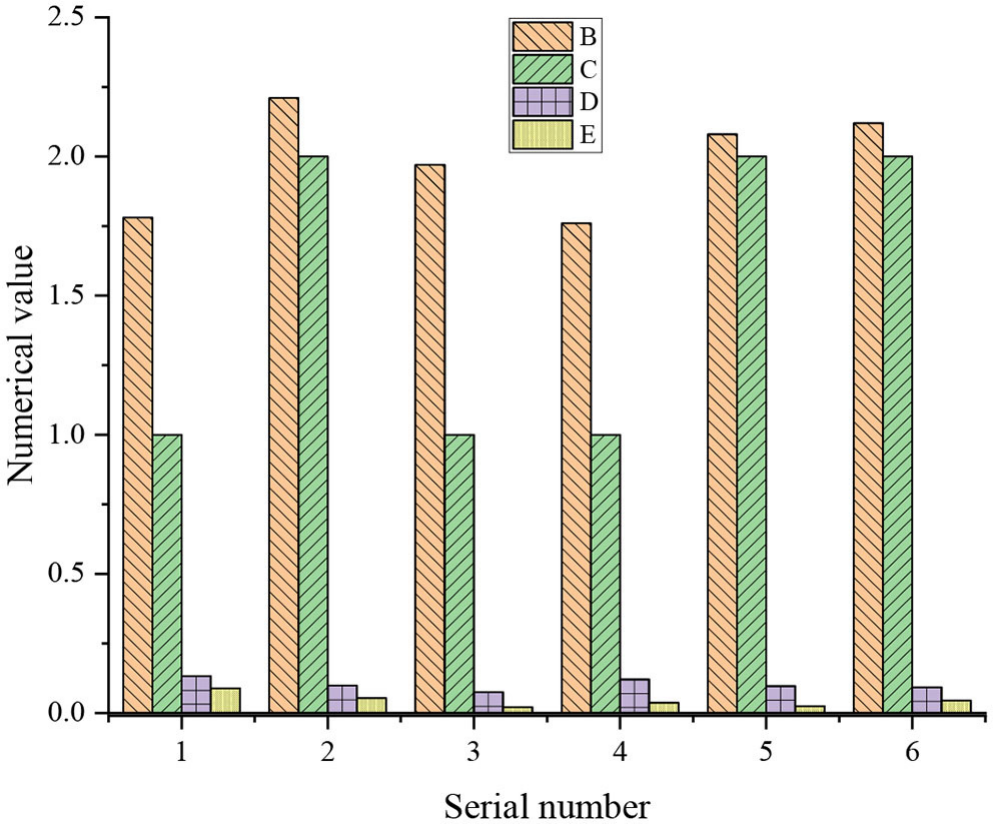
Prediction results of BPNN on mental problems (B is prediction output, C is standard value, D is absolute error, and E is relative error).

The test data are input into the established neural network model for the simulation test. After several groups of data simulation tests, it can be concluded that the error of each group of prediction results is relatively small, which meets the requirements. This shows that the model can predict the problem of the studied sample status. Figure 5 displays the prediction results.

Figure 5 shows that the real and relative errors of the proposed BPNN model are controlled within the range of 0.01 for the prediction results of six different data factors. It indicates that the BPNN model can predict the mental problems of children in plight within a certain range, with high prediction accuracy and small error value, and the prediction model is feasible.

### The Tests of Structural Equation Models

#### Goodness-Of-Fit Test

The SPSS and AMOS are adopted to conduct the path analysis of models, and the likelihood ratio (LR) is selected to test the goodness-of-fit (GFI) of the structural equation. The standard is that if the sum of all items in the tested models is more approximate to that of the saturated model, the GFI will be higher. Table 7 displays the GIF indices.

**Table 7:** Goodness-of-fit test.

	**Evaluation index**	**Index meaning**	**Recommended value**	**Results**
Absolute fitting index	CMIN/DF	Chi-square goodness of fit test	Between 1–3	2.623
	GFI	Goodness-of-fit index	>0.9	0.969
	AGFI	Adjusted goodness-of-fit index	>0.9	0.944
Relative fitting index	NFI	Norm-fitting index	>0.9	0.916
	IFI	Increasing fitting index	>0.9	0.946
	TLI	Tucker-lewins index	>0.9	0.918
	CFI	Comparative fit index	>0.9	0.945
Parsimony adjusted measures	PNFI	Parsimony-adjusted NFI	>0.5	0.611
	PCFI	Parsimony-adjusted CFI	>0.5	0.630

The goodness-of-fit in Table 6 suggests that all the fit indices are within the range of the proposed value, proving that the fitting results of the model are satisfying. Table 8 displays the results of further hypothesis tests.

**Table 8:** Hypothesis test results.

			**Estimate**	**S.E**.	**C.R**.	**P**
Self-efficacy	< –	Attachment	0.036	0.015	2.354	^*^
Self-efficacy	< –	Mental anxiety	−0.025	0.072	−0.344	0.731
Resilience	< –	Self-efficacy	−0.623	0.290	−2.148	^*^
Resilience	< –	Attachment	−0.602	0.098	−6.121	^***^
Resilience	< –	Mental anxiety	−0.714	0.270	−2.644	^**^
Stress response efficacy_1	< –	Self-efficacy	1.000			
Strive for self efficacy_2	< –	Self-efficacy	1.764	0.499	3.535	^***^
Study anxiety scored_1	< –	Mental anxiety	1.000			
Social anxiety_1	< –	Mental anxiety	1.638	0.193	8.467	^***^
Impulsive tendency_1	< –	Mental anxiety	0.926	0.129	7.200	^***^
Isolation tendency_1	< –	Mental anxiety	1.208	0.142	8.526	^***^
Inner strength_1	< –	Resilience	1.000			
Supported strength_1	< –	Resilience	1.084	0.095	11.361	^***^
Anxiety_1	< –	Attachment	1.000			
Escaping_1	< –	Attachment	1.104	0.144	7.688	^***^

* *means P < 0.05*,

** *means P < 0.01*,

*** *means P < 0.001 (Our study calculated the result to three decimal places)*.

Table 7 reveals that attachment directly affects resilience, and *P* < 0.001, so that Hypothesis 1 stands. Mental anxiety directly affects resilience, and *P* < 0.01; thus, Hypothesis 2' also stands. Self-efficacy has significant direct effects on resilience, and *P* < 0.05, so that Hypothesis 3 stands. The attachment has significant direct effects on resilience, and *P* < 0.05, so that self-efficacy may have mediating effects on the relationship between attachment and resilience. Then, Hypothesis 4 may stand. However, the direct effects of mental anxiety on self-efficacy have not passed the test, and *P* > 0.05, so that Hypothesis 5' has not passed the test. The mediating effects of self-efficacy on the relationship between mental anxiety and resilience may not exist. Hence, the test of mediating effects is further conducted.

#### The Test of Mediating Effects

The mediating effect test adopts Bootstrap Method, which takes the samples as a totality. If there is a totality with a sample size of N, sampling with replacement (selecting one case, putting it back, and then selecting another) is conducted until the number of selected cases is equal to N. These N cases make one sample.

To repeat the mentioned process by k times, k samples should be obtained. Based on each sample, an estimated value of mediating effects can be calculated, thus gaining a sampling distribution composed of the product of k coefficients. In this way, the confidence interval of the product of coefficients is further obtained. Generally, the Bootstrap method requires that the sampling time k is no less than 1,000. The sampling distribution is obtained after repeatedly selecting samples by 5,000 times. Table 9 presents the confidence interval of the product of coefficients.

**Table 9:** The upper limit and lower limit of bias-correction confidence interval.

	**Attachment**		**Mental anxiety**		**Self-efficacy**		**Resilience**	
	**Upper limit**	**Lower limit**	**Upper limit**	**Lower limit**	**Upper limit**	**Lower limit**	**Upper limit**	**Lower limit**
Self-efficacy	0.000	0.000	0.000	0.000	0.000	0.000	0.000	0.000
Resilience	−0.002	−0.060	0.203	−0.069	0.000	0.000	0.000	0.000
Escaping_1	0.000	0.000	0.000	0.000	0.000	0.000	0.000	0.000
Anxiety_1	0.000	0.000	0.000	0.000	0.000	0.000	0.000	0.000
Inner strength_1	−0.494	−1.024	−0.149	−1.363	0.119	−1.295	0.000	0.000
Supported strength_1	−0.477	−0.919	−0.126	−1.370	0.074	−1.308	0.000	0.000
Isolation tendency scored_1	0.000	0.000	0.000	0.000	0.000	0.000	0.000	0.000
Impulsive tendency scored_1	0.000	0.000	0.000	0.000	0.000	0.000	0.000	0.000
Social anxiety scored_1	0.000	0.000	0.000	0.000	0.000	0.000	0.000	0.000
Study anxiety scored_1	0.000	0.000	0.000	0.000	0.000	0.000	0.000	0.000
Strive for self efficacy	0.118	0.010	0.219	−0.296	0.000	0.000	0.000	0.000
Stress response efficacy_1	0.073	0.012	0.124	−0.210	0.000	0.000	0.000	0.000

The upper and lower limits of the bias-correction confidence interval are observed. The results reveal that the lower limits of indirect effects of attachment and mental anxiety on resilience are −0.060 and −0.069, respectively, and the upper limits are −0.002 and 0.203, respectively. This shows: (a) the bias-correction confidence interval of attachment and resilience does not include 0, so that attachment has significant mediating effects on resilience through self-efficacy; (b) the bias-correction confidence interval of mental anxiety and resilience includes 0 so that mental anxiety has insignificant mediating effects on resilience through self-efficacy.

### The Correction of Model and Conclusion

#### The Correction of Model

**Figure 6:**
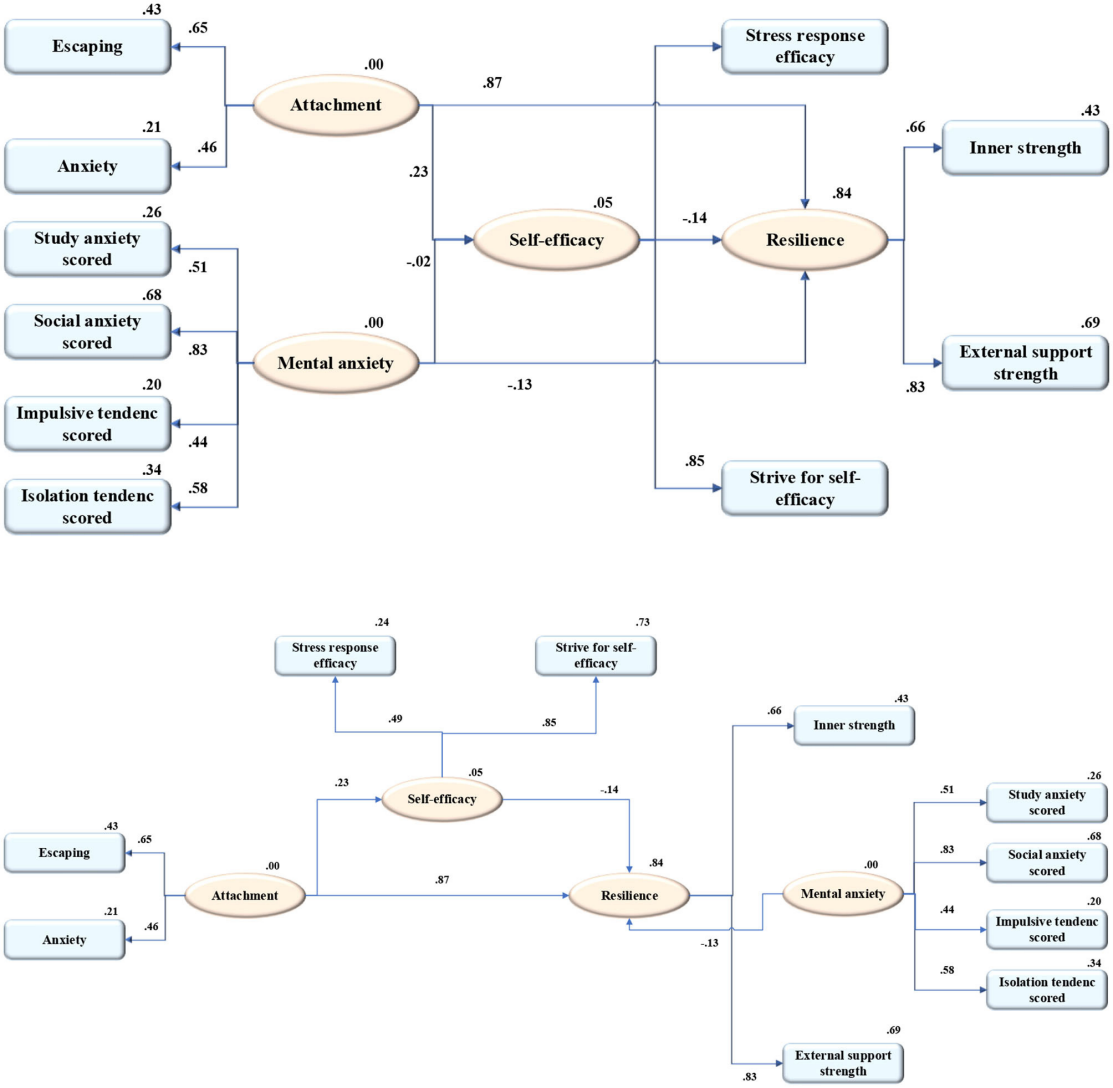
The corrected model of resilience micro-structure.

According to the test results of the hypothesis model (Figure 2), Hypothesis H5' has not been verified, while Hypotheses H1, H2', H3, and H4 have been verified. Therefore, it is necessary to modify the original model based on the test results, as shown in Figure 6.

## Discussion

First, the structural equation model is used to study the microstructure of poor children's mental elasticity, and to explore the structural relationship and functional path between children's mental elasticity and the self-efficacy of their mental health, psychological anxiety, and attachment. The results show that attachment, self-efficacy, and psychological anxiety have a significant direct impact on mental resilience. Self-efficacy has a mediating effect between attachment and resilience, but the mediating effect between the symptoms of psychological anxiety and resilience is not significant. Self-efficacy, psychological anxiety, and attachment have relatively independent contributions to children's mental elasticity. Children's self-efficacy is conducive to dealing with the supporting effect of external relationship resources. Psychological anxiety is a risk response mechanism to stress in the environment. Attachment can regulate resilience by providing security-based effects and positive coping styles. The above three factors jointly promote and develop children's mental elasticity. It can be concluded that the proposed structural equation model can effectively analyze the microstructure of mental elasticity of children in plight.

Then, children's mental problems are predicted by selecting relevant factors and using BPNN model. The results show that the prediction error of BPNN is 65.4% lower than the widely used SVR model, and BPNN has a better prediction performance. Moreover, the problem analysis efficiency of artificial intelligence technology is much higher than that of the artificial analysis, which will help to improve the overall efficiency and the quality of children's mental problem analysis.

The discussion on the questionnaire results is as follows.

(1) The resilience structures of self-efficacy, mental anxiety, and attachment are relatively independent and stable.

Children's self-efficacy, mental health, or symptoms, and attachment with adults are often used as the predictive indices for children's resilience development. Regarding the structural relations between these three factors and resilience, the mental anxiety, attachment, and self-efficacy of children in plight make independent contributions to their resilience level. Only self-efficacy has some mediating effects on the relationship between the resilience and attachment of children in plight. Hence, it makes sense to use the structures of these three factors in evaluating and predicting resilience in previous studies.

(2) Mental anxiety is a risk response mechanism to stress and difficulty in the environment.

There are also resilience studies that define resilience by taking mental health as a result of being resilient (Fellmeth et al., 2018). However, the results reveal that among the mental health factors directly related to resilience, only the scores of learning anxiety, anxiety toward others, impulse tendency, and autistic tendency can be put in the equation of regression. Thus, factors logically related to resilience consist of the anxious performance of children facing learning tasks and stress from interpersonal interaction, self-controlling ability in self-management, and the autistic tendency, resulting from subjective perception due to bad interpersonal interaction (Masten et al., 2006). However, for children in the plight to test whether their resilience is good, only mental anxiety-related factors are the response mechanism for individuals under pressure in adversity. Therefore, it has practical significance to consider these four factors related to mental anxiety as predictive indicators of resilience evaluation.

(3) Self-efficacy helps respond to the supportive effects of external resources.

The self-efficacy of children in plight has prominent predictive effects on resilience. Self-efficacy is more common in the studies on vulnerable children's mental health, often seen as the mediator variable of children's sense of happiness, behavioral problems, and academic achievement. It is closely related to children's self-respect, self-confidence, self-identification, and sense of social support. The results reveal that the positive self-efficacy of children in plight and their stress-coping efficacy both have positive predictive effects on their adaptive development. When they think they can deal with current difficulties through their efforts, such positive expectation and evaluation of their capacity can effectively predict their resilience functions. Moreover, the self-efficacy of children in adversity has notable mediating effects on attachment and resilience, suggesting that the avoidance or anxiety model in their attachment relationship can negatively influence adaptation results through self-efficacy. Children's high self-efficacy can effectively buffer anxiety or have adverse effects on avoidant attachment. Therefore, children's resilience level can be improved by cultivating the self-efficacy of children in plight.

Self-efficacy helps respond to the supportive effects of external resources. External resources can be divided into relational resources and instrumental resources. Emotional support mainly manifests relational resources to children, like encouragement, care, and companionship in adversity. Instrumental resources are manifested by economic support, material help, instruction, and suggestions regarding children's specific difficulties. High self-efficacy can help children effectively identify external supportive resources and make use of resources to cope with difficult situations, playing a protective role in children's resilience development. Self-efficacy's mediating effects on attachment are presented as a higher sense of safety and a better ability to express one's emotions, having a secure base effect on active exploration for children in adversity and cultivating children's positive coping ability and the development of their initiative.

(4) The regulatory functions of attachment.

As a regulatory mechanism of children's near-end supportive environment, attachment can affect the regulatory process of resilience through two approaches, providing a secure base effect and positive coping. First, the security base effect of attachment can directly affect resilience development. A securely attached relationship provides children in plight a comfortable and secure shelter; children can go back to the security base to protect themselves after identifying a crisis. An insecurely attached relationship is unable to provide such a security base, which will hinder children's exploratory behaviors in the new environment, thus suppressing their resilience.

Meanwhile, it will be easy for children with secure attachment to develop an excellent internal working model. Such children can get a sense of security and stability in more environments, thereby getting more comprehensive protective environments. Besides, they will quickly develop a confident personality and exploratory behaviors, and a securely attached relationship can help children seek positive coping methods under threat. In this way, the successful experience will improve their self-efficacy and indirectly affect their resilience.

In conclusion, the resilience process of children in plight is the result of the interaction between internal and external environments, which is a dynamic and complex process. In such a complicated context, helping children in plight establish a securely attached relationship and focusing on the conductive education on their self-efficacy and mental anxiety; autistic and impulsive behaviors can significantly help the adaptive development of their social psychology. Moreover, the three factors mentioned above can jointly help improve and develop the resilience of children.

## Conclusion

Deep learning algorithms and questionnaires are used to evaluate the mental problems of children in plight, so as to explore the microstructure of mental elasticity and resilience of children in plight. First, the microstructure of mental elasticity of children in plight is systematically studied by constructing the structural equation model. Then, the mental problems of children in plight are predicted based on the BPNN model. Finally, the performance of the model is verified by using real datasets. The experimental results show that the average prediction errors of the BPNN model and SVR model are 1.87 and 5.4, respectively. The error of BPNN is 65.4% lower than SVR and BPNN has better prediction performance. The prediction results on the test set show that the actual and relative errors of the BPNN model are controlled in the range of 0.01, and the prediction accuracy is high. The fitting indices of the structural equation model are within the recommended range, and the results are satisfactory. The results of the questionnaire analysis show that attachment, self-efficacy, and psychological anxiety have a significant direct impact on mental elasticity. The research limitation is that the collection range of data samples is concentrated in Northwest China, and it is unknown whether it is suitable for studies outside this range. Future work can focus on expanding the scope of data collection and further improving the model. This exploration aims to conduct a micro investigation on the relationship among the three core variables (attachment, self-efficacy, and mental health) in the resilience research of children in plight, and analyze their resilience, so as to provide a theoretical basis for resilience intervention design of vulnerable groups.

## Data Availability Statement

The raw data supporting the conclusions of this article will be made available by the authors, without undue reservation.

## Ethics Statement

The studies involving human participants were reviewed and approved by Xi'an Jiaotong University Ethics Committee. The patients/participants provided their written informed consent to participate in this study. Written informed consent was obtained from the individual(s) for the publication of any potentially identifiable images or data included in this article.

## Author Contributions

All authors listed have made a substantial, direct, and intellectual contribution to the work and approved it for publication.

## Conflict of Interest

The authors declare that the research was conducted in the absence of any commercial or financial relationships that could be construed as a potential conflict of interest.

## Publisher's Note

All claims expressed in this article are solely those of the authors and do not necessarily represent those of their affiliated organizations, or those of the publisher, the editors and the reviewers. Any product that may be evaluated in this article, or claim that may be made by its manufacturer, is not guaranteed or endorsed by the publisher.
